# A Review of the Effect of Injected Dextranomer/Hyaluronic Acid Copolymer Volume on Reflux Correction Following Endoscopic Injection

**DOI:** 10.1155/2008/579370

**Published:** 2008-09-29

**Authors:** Sumit Dave, Darius J. Bägli

**Affiliations:** ^1^Division of Urology, Children's Hospital, University of Western Ontario, London, ON, Canada N6A5W9; ^2^Division of Urology, The Hospital for Sick Children, University of Toronto, 555 University Avenue, Toronto, ON, Canada M5G 1X8

## Abstract

The current literature suggests that multiple variables affect vesicoureteric reflux (VUR) resolution rates following dextranomer/hyaluronic acid copolymer (Dx/HA) injection. This article reviews the evidence pertaining to the effect of injected Dx/HA volume on success rates following endoscopic correction. Lack of prospective studies which use injected volume as a continuous variable coupled with a nonstandardized injection technique and endpoint hinders the ability to reach a definite conclusion.

## 1. INTRODUCTION

The approval of dextranomer hyaluronic acid copolymer (Dx/HA) by the FDA in 2001, coupled with its safety and ease of injection, has led to a rapid increase in its use for treating vesicoureteral reflux (VUR) [1]. This has been accompanied by a reevaluation of the treatment philosophy for VUR, promulgated by both a change in physician preference and driven by parents who are opting for endoscopic correction over long-term follow up and antibiotic prophylaxis [2, 3]. However, in the era where basic concepts about VUR and its role in UTI and renal scarring continue to evolve, the mere availability of a minimally invasive approach cannot, in of itself, immediately justify the adoption of changed indications for VUR correction. Moreover, despite the high success rates shown in some large series, endoscopic VUR correction using Dx/HA has not yet achieved the success rates following open surgery [4]. It is, therefore, imperative that factors associated with success following Dx/HA injection are identified in order to improve surgical outcomes, gain insight into potential mechanisms which underlie success as well as failure, and enable better patient selection and preoperative counseling (Table 1).

The current paper reviews the impact of injected Dx/HA volume on primary VUR correction rates. Studies analyzing this variable are discussed along with the findings of a recent multivariable analysis conducted at our institution.

## 2. EFFECT OF INJECTION TECHNIQUE ON THE VOLUME OF Dx/HA

The known principles for VUR correction are derived from dissections dating back to the description of the physiologic submucosal tunnel by Paquin in 1950s, which defined the mechanistic basis of open surgical procedures to correct VUR [5]. By extrapolation, the goal of endoscopic injection is to create an effective valvular surrogate by providing submucosal support for the entire length of whatever portion of the refluxing ureter, that is, transvesical. This is achieved by accurate injection of sufficient amount of the bulking agent in a correct plane. The hydrodistension implantation technique (HIT) popularized by Kirsch and subsequently modified to a double HIT procedure has highlighted the importance of hydrodistension in enabling an intraureteric injection to target support to the entire intravesical ureter [6]. This technique was based on the initial description by Chertin et al. for injection therapy in children with high-grade VUR [7]. As opposed to the classical STING (subtrigonal injection) technique, which aims at achieving a good mound at the ureteral orifice, the HIT tends to involve higher volumes of injection as it aims to support the entire ureteric length. Moreover, obliteration of any further hydrodistension of the ureteral orifice is the endpoint in this technique rather than a good mound. Clearly, establishing this endpoint may further lead to higher injection volumes. Therefore, reported volumes of injection in all studies should be interpreted with caution, and both technique and volume should be studied as distinct variables in a multivariable analysis.

## 3. EFFECT OF INJECTED Dx/HA VOLUME ON OUTCOME

The mean injected volume of Dx/HA injected in all series reported to date varies between 0.2 mL to >1 mL [2, 3, 6, 8–16]. The impact of injected volume on success is variable (Table 2). Kirsch et al. found no statistical difference in injected volume between successes and failures using a mean of 0.83 mL in 459 ureters [8]. In a follow up study using a mean volume of 0.9 mL, the same authors demonstrated a positive impact of increasing experience as well as injected volume, with improved success rates from 60 to 74% [6]. The third variable, which then prompted a further improvement in the success rate to 89%, was the use of the “modified STING” or HIT. The HIT technique involves placing the needle into the mid to distal ureteral tunnel itself at the 6-o’ clock position and watching the entire tunnel coapt as the injection progresses. In contrast, the traditional STING technique, judged by both the mechanism and endpoint of injection (a mound at the ureteral orifice alone, not involving the intravesical ureter, and in effect creating a surrogate nipple valve rather than flap valve mechanism at the ureteral orifice to prevent VUR) would presumably require a relatively lesser injected volume. Though not highlighted in the paper, the injected volumes were indeed higher in this subset of patients (1–1.5 mL), compared to the STING group.

In contrast, in two subsequent studies where mean injected volumes of Dx/HA were ≥0.8 mL, no correlation with VUR correction was noted [9, 10]. Lavelle et al. reported that the average injected volume was 0.84 mL in those with successful VUR correction when compared with 0.94 mL in failures (*p* = NS) [9]. Mound morphology was the only statistically significant predictor of success; 87% of ureters that showed a “volcano” configuration were corrected as opposed to only 53% in those with an “alternate” morphology. Although Routh et al. did not demonstrate an effect of injected volume in their study, the authors acknowledged that their injection volume had increased over time based on the positive experience of other authors [10].

Yucel et al. performed a multivariable analysis of their experience with Dx/HA injection and showed that an injected volume of <0.5 mL was significantly associated with success as compared to a volume >0.5 mL [11]. The overall reflux correction was 70% by patients and 78% by ureters (mean VUR grade 2.6) as compared to 89% and 92%, respectively (mean VUR grade 2.6), in the study by Kirsch et al. [6]. Similar to the findings of Lavelle et al., this study showed that mound morphology was the most important indicator of VUR correction. The authors speculated that a higher volume of Dx/HA implied a technically more difficult injection resulting in a poorer outcome. No evidence was provided to support this conjecture. Moreover, it is unlikely that all injections in the HIT series by Kirsch et al. were uniformly more difficult to alone account for greater injected volumes. As stated above, a priori performance of a double HIT injection is likely to require more injected material. Alternatively, the findings of Yucel et al. may reflect that the analysis was based on a cutoff close to their mean injected volume, rather than treating the injected volume as a continuous variable.

Another multivariable analysis published in 2007 attempted to look at the effect of volume using a 1 mL cutoff. Routh et al. treated 301 patients (453 ureters) with VUR using an average 0.93 mL Dx/HA with a 75.5% success rate by ureters [12]. The authors noted that preoperative VUR grade and the operating surgeon were significant predictors of outcome. The technique of injection (HIT versus STING) was significant on a univariate analysis but only showed a trend toward significance for HIT on a multivariable analysis (*P* = .056). However, with respect to volume, no difference in success rates was noted when injected Dx/HA volume was analyzed as a cutoff of <1 mL or >1 mL. It is possible that arbitrarily choosing a 1 mL cutoff volume may have missed an actual significant cutoff volume, thereby, failing to detect any volume effect. Moreover, as rightly pointed out by the authors, there is also a possibility that the positive effect of higher volume is nullified by the fact that higher volumes are more likely to be used for higher grades of VUR.

We performed a retrospective review of 126 consecutive patients with primary VUR (196 refluxing ureters) who underwent injection for febrile urinary tract infections (UTI) to identify factors associated with success following Dx/HA injection [13]. Endoscopic injection was performed using both the STING and the HIT techniques in this series though neither were prospectively planned in any patient nor systematically varied over the course of the series. Success was defined as resolution of VUR after first injection on postoperative VCUG performed 3 months following endoscopic treatment. Univariate and multivariate regression analysis were performed on the following variables: age at surgery, gender, laterality, time between presentation and surgery, preoperative VUR grade, surgeon experience, lower urinary tract symptoms (LUTS), and volume of Dx/HA injection. Statistical analysis was performed with SPSS version 13.0 software (SPSS Inc., Chicago, Ill, USA), with *P*-values less than .05 considered statistically significant.

**Figure 1:**
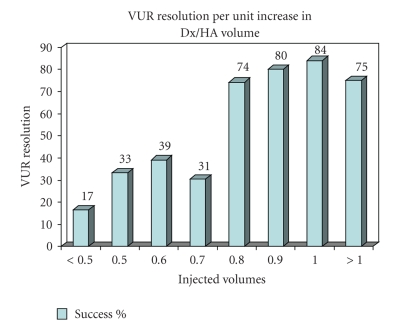
VUR correction rates for each 0.1 mL increment in injected Dx/HA volume.

By renal unit, VUR grades were as follows: I in 7(3.5%), II in 53(27%), III in 91(46.4%), IV in 30(15.3%), and V in 15(7.6%), with a mean VUR grade of 3. Success rate after 1 injection was 50% by patient and 59.2% by ureter. Success rate by grade was 100% for grade I, 75% for grade II, 57% for grade III, 37% for grade IV, and 46% for grade V. Mean injected volume was 0.9 ± 0.27 mL in those who had a successful injection versus 0.67 ± 0.24 mL in those who failed (*P* < .001). Success after 1 injection was 78.9% using ≥0.8 mL Dx/HA compared to 31.7% with <0.8 mL. The mean Dx/HA volume increased from 0.75 ± 0.26 mL in the first 98 ureters treated to 0.87 ± 0.29 mL in the last 98 (*P* = .002), a change that was associated with a simultaneous improvement in the success rate for grade III VUR from 50 to 68%. This increase in injected volume was not prompted by an interim assessment of our results, though it can be speculated that it may be a reflection of a change in technique form the classical STING to the HIT (see above). However, there was no statistical difference in the mean injected volume for high- and low-grade VUR: I–II (0.82 ± 0.29 mL) versus III–IV (0.78 ± 0.26 mL), indicating that grade did not influence injection volume across the series. The success rates for each 0.1 mL increase in injected Dx/HA volume is plotted in Figure 1. Our analysis showed that for each 0.1 mL increment in the injected Dx/HA volume, a statistically significant improvement in success rate was observed when compared to correction achieved below that cutoff volume. This volume effect persisted up to a maximum of 1 mL injected beyond which no further increase in success rate was observed (see discussion of arbitrary choice of cutoff volume in [12] above). Multivariable analysis confirmed that higher Dx/HA volume (*P* = .001), lower preoperative grade (*P* = .013), surgeon experience (*P* = .025), and treatment of LUTS (*P* = .009) were all independently associated with successful correction of VUR.

## 4. CAN INJECTED VOLUME IN ASSOCIATION WITH OBLITERATION OF HYDRODISTENSION BE USED IN COMBINATION TO PREDICT SUCCESS?

The use of mound morphology as the injection progresses as a predictor of VUR resolution is fraught with some inherent drawbacks. What defines a “good” mound is a subjective measure much like the subjectivity of the “good urethral plate” in hypospadias surgery; both are qualitative, difficult to define, and are based on surgeon experience. Secondly, the mound is a 2-dimensional view of the effect of the injection at the ureteric orifice, but gives no indication of the support achieved, if any, along the entire intra vesical ureter. In addition, the mound at injection may not be the mound at the time of reassessment by a VCUG at 3 months. There is a well-documented 19% decrease in the injected Dx/HA bolus over 3 months [8]. This volume reduction occurs because the dextranomer microspheres constitute 50% of the volume in Dx/HA and their hydrolysis overtime will alter the mound morphology, likely shrinking it somewhat, notwithstanding the stabilizing effect of collagen ingrowth [17]. This coupled with a risk of bolus migration would mean that the surgeon could use mound morphology as a predictor of VUR correction *at the time of injection* but this endpoint may not be a stable indicator of longer term success. In studies which showed the effect of a “favorable” mound morphology on outcome, VUR resolved in 53% of ureters in Lavelle’s series and in 36% in the study by Yucel et al. [11]. Moreover, up to 12% of “good” mounds can have persistent VUR following Dx/HA injection [18].

These studies all share the inherent limitations of nonrandomized retrospective reviews. In the present paper, this primarily involves failure to identify and include of all confounding variables which could impact the results. For example, one of the criticisms of our study is that the technique of injection (HIT versus STING) was not analyzed. Moreover, the very indications for treatment of the reflux vary from study to study, along with the severity of VUR further confounding the results and their comparison with other studies.

## 5. CONCLUSIONS

There are several factors which may predict successful VUR correction following Dx/HA injection. Our study revealed the presence of a direct association between injected volume and VUR correction, by treating volume as a continuous variable, even while controlling for other variables, highlighting its importance as a true success modifier. The injected volume of Dx/HA is a factor, which to a degree under the direct control of the surgeon. Given the exigencies of materials cost, and the expectation on surgeons to use available medical resources responsibly, without a clear demonstration of the effect of volume on results, the surgeon is to a certain extent hesitant to use only a small portion of a second Dx/HA syringe, beyond the 0.8–1 mL available for injection in the standard commercially available syringe. Based on our experience, we now adopt a more aggressive approach in injecting a minimum of 0.8 mL irrespective of the grade of VUR and ensure obliteration of hydrodistension at the end of injection. From a cost stand point, an injection failure definitely involves higher costs and, therefore, it is reasonable to use a higher volume at the initial attempt to improve success rates. Syringes with slightly greater volumes of 1.2–1.4 mL, should they become available in the future, may provide greater treatment flexibility in this regard. Finally, though endoscopic injection for VUR is generally accepted as a simple procedure, the importance of technique and experience are evident in most studies. Further prospective studies which include all variables, and which possibly perform hydrodistension in a standardized manner, need to be conducted to identify factors which can be used for patient counselling, and increase success rates to those won by open correction of VUR.

## Figures and Tables

**Table 1:** Reported variables associated with VUR correction using Dx/HA.

Variables associated with VUR correction following Dx/HA
injection
(1) Mound morphology
(2) Grade of VUR
(3) Surgeon experience/learning curve
(4) Injection technique
(5) Volume of Dx/HA
(6) Absence of ureteric dilatation
(7) Location of ureteral orifice (degree of lateral ectopia)
(8) Age of patient
(9) Resident participation
(10) Fewer needle insertions
(11) Absence or correction of lower urinary tract symptoms

**Table 2:** Studies investigating the effect of injected Dx/HA volume on VUR correction.

Series	Refluxing units; mean	Mean	Volume injected (mL)	Method of statistical	Success by	Effect of volume of
	Dx/HA volume (mL)	grade	success/failures	analysis	grade (%)	injected Dx/HA
Kirsch 2003	292	2.6	S: 0.9 ± 0.3	Univariate	I 90; II 82;	NS
	0.83 ± 0.03		F: 0.9 ± 0.2		III 73; IV 65	

Kirsch 2004	119		S: 1.0		I 100; II 90;	Higher
modified	>0.9	2.8	F: 1.5	Univariate	III 91; IV 89	volume
STING						significant

Lavelle	80	NA	S: 0.8	Univariate	I 82; II 84;	NS
2005	NA		F: 0.9		III 77; IV 73	

Routh	225 pts	2.4	S: 0.8 (0.4–2.0)	Univariate	I 63; II 72; III 57;	NS
2006	0.8 (0.3–2.0)		F: 0.8 (0.3–1.8)		IV 14 (By patients)	

		2.6	NA	Studied as categorical	I 100; II 83;	Lower ( <0.5)
Yucel	259			variable with cutoff	III 73;	volume
2007	0.54 ± 0.2			</ >0.5 mL using	IV 53,	significant
				multivariable analysis	V 29	

		2.3	NA	Studied as categorical	I 83;	NS
Routh	453			variable with cut off	II 82;	
2007	0.93 (0.2–3.5)			</ >1 mL using	III 66;	
				multivariable analysis	IV 53	

		3			I 100,	Higher
Dave 2007	196		S: 0.9 ± 0.2	Studied as continuous	II 75;	volume
(Accepted J	0.8 ± 0.03		F: 0.6 ± 0.2	variable using	III 57;	significant on
Urol)				multivariable analysis	IV 37;	multivariable
					V 46	analysis
